# Mr. Boyle's Treatise on Syphilis, &c.

**Published:** 1824-06-01

**Authors:** 


*A Treatise on Syphilis; exhibiting the Advantages of large Doses of the Submuriate of Mercury in the Cure of that Disease: also, an Inquiry into the Modus Operandi of Mercury. Illustrated by Experiments.* By JAMES BOYLE, Esq. one of the Surgeons to the United Institution of London and Westminster for the Treatment of Lying-in Women, and Children, &c. &c. Octavo, pp. 166. London, March, 1824.

THE question respecting the *possibility* of curing syphilis without mercury appears now to be nearly at rest, by being answered in the affirmative. But, very few, we imagine, avail themselves of this piece of knowledge; and of those few who do, some have had cause to repent their experiments, by losing their patients, and with them some part of their reputation. We are every day more and more convinced of the general superiority of the mercurial over the non-mercurial practice in the venereal disease, though exceptions will occur, from time to time, where it will be prudent to suspend or omit the specific, and trust to low living, quietude, and gentle evacuations for the eradication of the disease. The question to which we dedicate the present short article is this—*is the slow introduction of mercury into the system, for the cure of primary sores, (as now practised,) more effectual or safe than a rapid introduction of the same?* We have not sufficient experience on this point ourselves to give a decided opinion; but we will furnish our readers with some evidence, of a very strong kind, which rests on the experience of others.

In the month of March, 1818, Mr. Boyle, author of the work before us, published in the Medico-Chirurgical Journal, (Monthly Series,) a letter on the subject of curing primary sores in an expeditious manner by large doses of mercury, adducing cases to prove the eligibility of the plan. Mr. Boyle there observes that, in many cases of syphilis which he had seen, the patients continued under a mercurial course for several weeks, without experiencing ptyalism, or any favourable change in the disease; "but when, from accidental circumstance, any of these patients happened to be confined to bed, in an apartment where the air was close, a violent salivation would suddenly break out, followed by the almost immediate healing of the chancres." Reflecting on this phenomenon, Mr. B. imagined that a single dose of mercury, if sufficiently large to induce ptyalism, might effect a cure in recent venereal infection. Being then in His Majesty's ship Prometheus, he had soon an opportunity of putting his theory to the test of experience, and the cases are detailed in the abovementioned journal, to which we must refer. We may state, however, that he found large doses of calomel neither produce violent salivation, nor violent purging. In the cases detailed, the mouth was sore about the third day, and the chancres were healed about the tenth. The dose of the calomel was generally a scruple, twice a day. The most rigid regimen was enjoined, with warmth and confinement.

In the month of May, 1818, Mr. Cunningham, a very able and experienced surgeon in the Royal Navy, and without any knowledge of Mr. Boyle's experiments, published a paper in the same journal, and upon the same subject—namely, "on the advantages of exhibiting submuriate of mercury, in scruple doses, in the cure of syphilis." He states that, for 12 years previously, he had been in the habit of treating a considerable number of cases in this way, as his journals at the Navy Medical Board would shew. He has published the details of several of these cases in the medical journal abovementioned, and to it we refer. Mr. Cunningham's practice was a scruple once or twice a day—the mercurial sore mouth coming on in two or three days, and the sores being healed in a few days afterwards.

The foregoing," says he, "which are a most faithful transcript from the original cases, I hope will be deemed quite sufficient to establish a striking testimony of the rapidity with which calomel, administered in scruple doses, impregnates the system, and consequently subverts the syphilitic virus. They prove, too, that the remedy may be employed in that proportion with perfect safety to the patient—for from many hundreds of such doses, which I have been constantly in the habit of giving since 1806, in the climate of this country, and in all seasons, not only for the cure of syphilis, but, with admirable benefit, in febrile and other affections, as dysentery, hepatitis, &c. I have never observed, in a single instance, any alarming, or even untoward effect."*

We now come to Mr. Boyle's autograph, published in the beginning of the present year, on the subject of syphilis, and more especially on this modification of treatment, strengthened by seven years' farther experience. As a preliminary argument in favour of the speedy administration of mercury, as contrasted with the usual slow process, our author introduces the following query.

"If, for example, two patients, under precisely the same circumstances, entered on means of cure at the same time; the one shall be put under a five weeks course of mercury, so conducted as to make the mouth sensibly affected at the end of that time, at which time also his sores or other venereal symptoms shall have disappeared. If again the other patient's mouth shall be affected in two days, his sores heal in four or five after, and the salivary glands continue in a state of excitation for six or eight days more:—I ask, which of these patients is likely to suffer most from the effects of the disease and the remedy? why assuredly, if we admit the venereal to be a disease capable of acting upon the constitution generally, we can have no hesitation in saying, the sooner that power is destroyed the less injurious will be its effects on that constitution; and the reverse of this, on procrastination." 103.

In support of the above, he quotes passages from John Hunter, Benjamin Bell, Mr. Geoghegan, &c. We shall offer one of the quotations from John Hunter.

"'Mercury should be given, if possible, so as to produce sensible effects upon some part of the body, and in the largest quantity that can be given within certain bounds; and that these sensible effects should

* Med. Chir. Journal, vol. v. p. 367.

be the means of determining how far the medicine may be pushed in order to have its best effects upon the disease without endangering the constitution. The practice here must vary according to circumstances; if the disease is in a violent degree, less regard must be had to the constitution, and the mercury is to be thrown-in in larger quantities; but if the disease be mild, it is not necessary to go beyond that rule, although it is better to keep up to it, in order to cure the disease the sooner.'" 107.

Mr. Boyle then goes on to the detail of numerous cases treated, since the year 1817, on the plan in question. To these cases we must refer the surgical reader, merely stating that they appear to bear the author out in his proposal for this modification of practice. Extracts of letters are also introduced from Mr. Bowen, Mr. Reid, and Mr. Cunningham, of the Royal Navy, all in corroboration of the same.

The work contains a great deal of curious and original observation, but the overstepped limits of our Review department compel us here to bring this short notice to a close—recommending to the attention of our surgical brethren the contents of Mr. Boyle's little volume.

